# An Effective COVID-19 Vaccine Needs to Engage T Cells

**DOI:** 10.3389/fimmu.2020.581807

**Published:** 2020-09-28

**Authors:** Karsten Sauer, Tim Harris

**Affiliations:** Repertoire Immune Medicines, Cambridge, MA, United States

**Keywords:** COVID−19, SARS—CoV-2, T cell, epitope, vaccine, neutralizing antibody, memory, pandemic

## Introduction

Ending the current COVID-19 pandemic and preventing recurrence requires the development of vaccines that provide long-lasting immunity to the causative virus SARS-CoV-2 and its emerging variants where B cell epitopes can be mutated (1–3). We argue that to achieve this, a vaccine must elicit CD4 and CD8 T cell immunity in addition to the production of neutralizing antibodies (nAB) by B cells. The rationale is based on the following findings for SARS-CoV-2 and the related SARS-CoV virus which caused the 2002/2003 SARS pandemic:

## Antibodies Alone May Not Protect Sufficiently

Most current vaccine efforts primarily aim to promote nAB production. However, significant evidence indicates that a robust antibody (AB) response alone is insufficient to avoid severe disease and might even promote it under certain poorly understood circumstances (4). While often critical for virus neutralization and disease control, B cell responses to the SARS-CoV-2 related virus SARS-CoV have been of limited duration and breadth (5–7). Although AB and nAB against SARS-CoV-2 are found in most cases of confirmed COVID-19 over time and can correlate inversely with viral load, their correlation with protection is unclear owing to a paucity of data and the use of heterogeneous serological assays with limited sensitivity and specificity (8). Signals in pre-COVID samples also indicate confounding cross-reactivities. In addition, AB need not protect, might worsen pathology, and high titers associated with more severe COVID-19 and worse outcomes in several studies—reminiscent of findings in SARS (4, 9, 10). Some unprotected ICU patients had SARS-CoV-2 specific AB, challenging their ability to protect (11). These findings point to a complex role of AB in COVID-19 that may not always be beneficial. It is also possible that mutational alteration of B cell epitopes could render emerging SARS-CoV-2 variants less sensitive to B cell engaging vaccines targeting earlier variants (2). Moreover, nAB titers declined to near baseline within 2–3 months during convalescence in many PCR-confirmed subjects particularly with mild disease or asymptomatic infection (12–14). Altogether, variable and often low nAB titers in convalescent patients, and in particular the very low titers or entire absence of nAB or AB against SARS-CoV-2 in up to 33% of recovered patients point to a critical role for other immune mechanisms in recovery from the disease (15–18).

## The Case for T Cells

Multiple lines of evidence support important roles for T cells in productive immune responses to COVID-19. In most SARS patients, B cell and nAB responses were relatively short lived (1–2 years) and prone to antigen escape, raising the possibility of re-infection. In contrast, T cell memory in survivors was long-lived (>6–17 years) (4–7, 19). It is well-known that T cells can engage antigen epitopes that are not targeted by B cells, including those derived from intracellular proteins, to provide broader protection which the virus can less easily circumvent through mutation (6). T cells are especially necessary to clear severe virus infections. Altogether, in addition to nAB, eliciting broad and long-lasting antiviral immunity requires the co-enrollment of CD4 and CD8 T cells and the generation of effective T cell memory (5, 6, 20–22).

The importance of T cells is further illustrated by the T cell lymphopenia and exhaustion or dysfunction in both SARS and COVID-19 which increase with disease severity (5, 16, 22–28). T cells clonally expand in COVID-19, and convalescence is associated with T cell recovery and memory formation. Convalescent patients contain SARS-CoV-2 reactive CD4 T cells (up to 100% of patients) and CD8 T cells (~70% of patients), including CD4 T_FH_ cells capable of providing help to B cells (29–32). Consistent with a role for T cells in helping B cells in COVID-19, virus-specific T and T_FH_ cell numbers correlated with nAB and AB titers and deceased COVID-19 patients lacked T_FH_ cells and germinal centers in their draining hilar lymph nodes correlated with reduced AB levels (32–37). T cell reactivities in recovered patients covered multiple SARS-CoV-2 proteins and particularly targeted immunodominant epitopes in the spike (S), membrane (M), and nucleoproteins (N), indicating a benefit for including these proteins in vaccine designs rather than only S as done in several current vaccines (32, 38). It has been shown that S-protein specific CD4 T cells correlate with virus-specific AB titers, but differing approaches and populations have yielded somewhat different antigen hierarchies and more detailed studies are needed to identify the most beneficial epitopes (39). Detection of SARS-CoV-2 cross-reactive CD8 and particularly CD4 T cells, probably elicited by endemic common cold causing coronaviruses, in 40–81% of unexposed individuals may contribute to the relative protection of most people against COVID-19 (16, 19, 29, 30, 37, 40). Further supporting T cell importance, *in silico* predictions and epidemiologic studies suggest that COVID-19 vulnerability may depend on the HLA haplotype of a person and its capacity to present SARS-CoV-2 epitopes to T cells. In Italy, prevalence of the potentially permissive alleles HLA-B^*^44 and C^*^01 correlates with COVID-19 spread (41, 42).

In SARS, both cytotoxic CD8 T cells and CD4 helper T cells were required for virus clearance (20). In COVID-19, several clinical studies have identified reduced CD8 T cells as an early prognostic indicator of severe or lethal disease and treatment efficacy. CD4 help is needed for effective anti-viral responses by both CD8 T cells and B cells, including nAB production (5, 29, 30, 43). In particular, airway memory CD4 T cells are critical for SARS-CoV clearance (6). Although SARS-CoV-2 reactive CD4 and CD8 T cells can be found in severe COVID-19 and can correlate with AB appearance and lower viral loads, CD4 T cells in severe disease are often dysfunctional or deregulated compared to mild COVID-19 (10, 23, 28, 30, 32, 37, 44, 45). Conversely, recovering patients had increased virus specific and general CD4 T_FH_ cells, and their SARS-CoV-2 reactive T cells expressed reduced inhibitory markers and elevated effector molecules (25, 34, 44, 46, 47).

Supporting the importance of T cells in controlling COVID-19, several studies reported durable T cell responses in convalescent patients even lacking virus-specific AB. One study found SARS-CoV-2 specific polyfunctional T cells with a stem-cell like memory phenotype in convalescent patients that could even be found in AB-seronegative family members and in individuals with a history of asymptomatic or mild COVID-19 (16). Another study found SARS-CoV-2 specific T cells but not AB in individuals that had been symptomatic within a week post-contact with COVID-19 infected relatives. These T cells persisted for at least 80 days and reached frequencies similar to those found in patients, which were much higher than the amounts in unexposed healthy donors and unlikely to reflect cross-reactivity with other coronaviruses (48). A third study found SARS-CoV-2 specific CD4 and CD8 T cell responses in 56% of AB-negative subjects (40). Thus, as seen in SARS and MERS, T cell immunity against SARS-CoV-2 can occur in the absence of humoral immunity, might even be more prevalent in certain populations, associates with recovery, can persist longer and might serve as a more sensitive biomarker for exposure (19, 48). The relative importance of humoral vs. T cell immunity for protection however, remains to be determined.

## T Cell Function Needs to Be Tuned Appropriately

Although T cells are required for effective virus control, excessive immune responses and a resulting cytokine storm can worsen disease and increase mortality in SARS and COVID-19. This might involve defects in immunosuppressive T_reg_ or γδ T cells, or the presence of “pathologic” CD4 T cells producing GM-CSF and IL-6 (5, 23, 49, 50). In SARS, severe disease was associated with increased virus-specific polyfunctional CD8 and CD4 T cells and T_H_2 cytokines (20). Encouragingly, strong T_H_2 skewing has not been seen in COVID-19 to date and two leading SARS-CoV-2 vaccine candidates elicited T_H_1 skewed responses in humans (4, 30, 31, 51, 52). Anecdotally, COVID-19 patients enter the hospital lymphopenic but begin to have increased respiratory difficulties as their lymphocyte counts start to recover. Consistent with this, SARS-CoV-2 reactive CD4 and CD8 T cells capable of producing effector and T_H_1 cytokines were found in patients with severe COVID (37). Another study found that SARS-CoV-2 specific T cells in acute moderate or severe COVID-19 were more activated and proliferating than those in convalescent patients, whose T cells had more memory-like phenotypes (16). A third study suggests increased proportions of SARS-CoV-2 reactive cytotoxic T_FH_ cells with dysfunctional/exhausted gene signatures and of clonally expanded cytotoxic CD4 T_H_ cells producing myeloid cell attracting chemokines; but under-represented SARS-CoV-2 reactive suppressive T_reg_ cells and polyfunctional T_H_1 and T_H_17 cells in severe vs. mild COVID-19 (44). Nevertheless, a clear cause-effect relationship between T cell phenotype and disease severity remains to be firmly established (39). Promoting T cell function promises improved virus clearance but may be detrimental in some patients, and a better understanding of the respective relevance of various T cell subsets and phenotypes for mild vs. severe disease is required (37).

In any case, a vaccine needs to promote desired effector, helper and memory T cell phenotypes and to tune T cell reactivity into a productive but safe “window,” while avoiding T_H_2 phenotypes, T_reg_ cell deregulation, and excessive T cell activation and exhaustion (39). To enable this, there is an urgent need to chart the time course, reactivities, and phenotypes of T cells against SARS-CoV-2 epitopes in healthy subjects and patients with different disease courses. Supporting a role for T cell reactivity and specificity in driving phenotype, a recent study found that in convalescent patients, SARS-CoV-2 S-protein specific CD4 T cells were skewed toward a circulating T_FH_ phenotype, whereas M- and N-protein specific CD4 T cells were skewed toward a T_H_1 or a T_H_1/T_H_17 profile (16). Another study found more multifunctional CD8 T cells targeting M and N than S in mild COVID-19 (32). Consistent with pathophysiological relevance of these observations, details of the SARS-CoV-2 T cell antigen hierarchy may differ with disease severity (39).

## A Need for Decoding Reactivities, Phenotypes, and Recognized Epitopes of SARS-CoV-2 Reactive T Cells

A recent review highlighted the importance of determining how SARS-CoV-2 impacts the T cell repertoire, and how the COVID-19 associated lymphopenia or disease predisposing comorbidities impact it (53). As discussed there, the importance of the T cell repertoire has long been recognized in other virus infections. In *Influenza* patients, the presence of specific T cell clones correlated with antiviral immunity, and an aging (thus more restricted) repertoire associated with increased infection. Influenza-epitope specific public T cell clonotypes that are shared between individuals have been identified (54). Such repertoire convergence is probably broadly relevant for virus infections, because T cell receptor (TCR) β profiling has shown convergent repertoire evolution in individuals infected with cytomegalovirus (CMV) or vaccinated against Yellow Fever (53, 55, 56). Public TCRs have also been used to identify smallpox vaccinated mice with > 99% accuracy (53, 57). This suggests that generalizable, convergent features of the T cell repertoire correlate with protection. Clearly the most convergent feature is T cell reactivity against certain antigenic epitopes. Hence it is possible that decoded antigen epitopes for convergent T cell clonotypes can be used for development of improved, T cell engaging vaccines against SARS-CoV-2. If the different phenotype skewing of T cells against different SARS-CoV-2 proteins confirms in other studies, an optimized choice of antigens or even epitopes might be able to instruct desired T cell phenotypes over undesired ones. Decoded T cell reactivities could also be used as sensitive correlates of protection, or to distinguish previously exposed individuals from unexposed ones and act as a biomarker of herd immunity even in AB seronegative individuals (16). Decoding matched T cell clonotypes, phenotypes and antigenic epitopes in COVID-19 patients will also answer the important questions as to which characteristics of the T cell repertoire explain the higher risk of the elderly, and how HLA genetic diversity contributes to the SARS-CoV-2 specific T cell repertoire and immune response (53). Such studies are now possible thanks to recent advances in single cell sequencing technologies combined with large-scale HLA tetramer or HLA reporter gene-based epitope library screening technologies (38, 58–66).

Indeed, several recent studies reported initial results from profiling B cell receptor (BCR) and TCR repertoires in COVID-19 patients (27, 45, 67, 68). Consistent with the data from other virus infections, they report convergent B cell clonotypic responses closely associated with SARS-CoV-2 AB. Somatic hypermutation analyses suggest a primary immune response involving naive B cells. Higher somatic hypermutation is associated with more severe disease, which also showed skewed BCR gene usage (27). Moreover, SARS-CoV-2 T cell responses were highly clonal in active disease and driven by TCR clusters shared between patients particularly after recovery, which showed characteristic clonotype trajectories over the disease course. Reduced T cell clonal expansion and skewed TCR gene usage in severe disease could indicate that different immunodominant antigen epitopes drive distinct T cell clonotypes and fates in mild vs. severe COVID-19 (27). A vaccine therefore may need to exclude B cell and T cell epitopes driving severe disease.

Additional recent studies have decoded blood T cell clonotypes and recognized antigen epitopes restricted by HLA class I or II in mostly convalescent COVID-19 patients and controls (19, 32, 38, 40, 67, 69, 70). Taken together, the studies show that anti-SARS-CoV-2 T cell responses target immunodominant epitopes broadly spread across the viral proteome mostly beyond the S protein, involve convergent and shared T cell clonotypes among patients and persist for several months post-recovery. Epitope localization outside regions with high mutational variation could suggest that T cell vaccine responses may not be prone to virus escape (69). An increased diversity but not intensity of SARS-CoV-2 T cell responses is associated with recovery from mild vs. severe disease (40). This may suggest that development of protective immunity requires recognition of multiple virus epitopes, arguing for including multiple SARS-CoV-2 antigens in vaccine design. However, in another study, responses appeared larger with a broader epitope coverage in severe patients (32, 39). Interestingly, several studies suggest shifted SARS-CoV-2 epitope hierarchies between COVID-19 patients and unexposed individuals harboring SARS-CoV-2 reactive T cells (19, 30, 32). In one study, unexposed individuals had cross-reactive T cells to 31% of identified HLA-I and 70% of HLA-II restricted epitopes (40). This may reflect previous infections with various betacoronaviruses or other crossreactivities whose physiological relevance remains to be elucidated.

These results revise the previously reported T cell antigen hierarchies dominated by S, M, and N to include ORF1 and other proteins. They emphasize the benefit of targeting T cells for development of broadly protective vaccines and highlight the need to include antigens beyond S, although broader studies covering more HLA haplotypes and including CD4 T cells are needed to identify the most promiscuous epitopes across large human populations, including N_81−120_ (19, 32, 39). The precise impact of virus reactive T cell clonotypes and their epitope specificity and phenotype on disease course, and their prognostic relevance remain to be elucidated. Pointing to the latter, profiling a subject's TCR repertoire worked as a diagnostic for past or current COVID-19 even in absence of virus-specific AB (67).

## Current Vaccine Candidates Engage T Cells to A Limited Extent

Several recent publications provide glimpses into how the most advanced current candidate SARS-CoV-2 vaccines engage T cells. All target the S-protein or its receptor-binding domain (RBD). In clinical trials, most candidates elicited robust nAB responses similar to or exceeding those in convalescent serum within the limited follow-up periods reported (51, 52, 71, 72). A human adenovirus based vaccine targeting S had limited immunogenicity due to high pre-existing anti-adenovirus nAB in many subjects (73, 74). Among all candidates, T cell responses were variable with frequencies of 100/10^6^-856/10^6^ IFNγ-producing, virus-specific peripheral blood derived CD4 and CD8 T cells in ELISPOT assays. A chimpanzee adenovirus based vaccine targeting the S protein elicited S-specific T cell responses lasting at least 56 days in many subjects (71). In contrast, the human adenovirus based vaccine elicited more limited virus-reactive T cells in ≤ 90% of subjects after 28 days (73, 74). One mRNA vaccine targeting the RBD caused RBD-specific CD8 T cell responses similar to, and T_H_1-skewed CD4 T cell responses exceeding memory responses to CMV, EBV, influenza & tetanus toxoid in > 80% of participants within 29 days, which correlated with nAB titers and varied among individuals (52). Similarly, another mRNA vaccine targeting S elicited T_H_1-skewed S-protein specific CD4 T cell but low CD8 T cell responses within 43 days in most subjects (51).

For all candidates, it remains unclear whether the T cell responses are high enough for robust and lasting protection. Because they only target S, all fail to leverage the majority of SARS-CoV-2 T cell epitopes that are clearly targeted in naturally infected individuals. Thus, a strong need remains for vaccines leveraging all T cell epitopes, and for large phase 3 trials to demonstrate durable efficacy in diverse human populations.

## Conclusion

Altogether, the data reviewed here point toward an important need to design COVID-19 vaccines which co-engage T cells in addition to B cells. They also highlight the benefit of decoding matched T cell reactivities, phenotypes and antigenic epitopes in the context of the major human HLA haplotypes for development of both vaccines and TCR based diagnostics for this devastating disease.

## Author Contributions

All authors conceived and wrote the manuscript together.

## Conflict of Interest

The authors declare that this study received funding from Repertoire Immune Medicines, Inc. The funder had the following involvement in the study: Both authors are full-time employees of Repertoire and have equity interest in it. Repertoire funded all work and provided equipment. Repertoire employees internally reviewed the manuscript, provided comments and cleared the manuscript for publication.
